# Molecular anatomy and pathogenic actions of *Helicobacter pylori* CagA that underpin gastric carcinogenesis

**DOI:** 10.1038/s41423-019-0339-5

**Published:** 2019-12-05

**Authors:** Atsushi Takahashi-Kanemitsu, Christopher T. Knight, Masanori Hatakeyama

**Affiliations:** Department of Microbiology, Graduate School of Medicine, The University of Tokyo, Tokyo, Japan

**Keywords:** *cagA*/CagA, *Helicobacter pylori*, SHP2, PAR1, inflammation, “hit-and-run” carcinogenesis, Gastric cancer, Cancer microenvironment

## Abstract

Chronic infection with *Helicobacter pylori cagA*-positive strains is the strongest risk factor for gastric cancer. The *cagA* gene product, CagA, is delivered into gastric epithelial cells via the bacterial type IV secretion system. Delivered CagA then undergoes tyrosine phosphorylation at the Glu-Pro-Ile-Tyr-Ala (EPIYA) motifs in its C-terminal region and acts as an oncogenic scaffold protein that physically interacts with multiple host signaling proteins in both tyrosine phosphorylation-dependent and -independent manners. Analysis of CagA using in vitro cultured gastric epithelial cells has indicated that the nonphysiological scaffolding actions of CagA cell-autonomously promote the malignant transformation of the cells by endowing the cells with multiple phenotypic cancer hallmarks: sustained proliferation, evasion of growth suppressors, invasiveness, resistance to cell death, and genomic instability. Transgenic expression of CagA in mice leads to in vivo oncogenic action of CagA without any overt inflammation. The in vivo oncogenic activity of CagA is further potentiated in the presence of chronic inflammation. Since *Helicobacter pylori* infection triggers a proinflammatory response in host cells, a feedforward stimulation loop that augments the oncogenic actions of CagA and inflammation is created in CagA-injected gastric mucosa. Given that *Helicobacter pylori* is no longer colonized in established gastric cancer lesions, the multistep nature of gastric cancer development should include a “hit-and-run” process of CagA action. Thus, acquisition of genetic and epigenetic alterations that compensate for CagA-directed cancer hallmarks may be required for completion of the “hit-and-run” process of gastric carcinogenesis.

## Introduction

Gastric cancer is the fifth most common malignancy and the third leading cause of cancer death, accounting for 819,000 deaths worldwide in 2018.^1^ While gastric cancer is diagnosed most frequently in developed nations, there exist large geographic variations in gastric cancer incidence.^1^ In Eastern Asian countries such as Japan, China, and Korea, the incidence is 8-fold greater than that in North America, accounting for more than half of the total gastric cancer incidence.^1^ Gastric cancer is also primarily a male-dominant cancer, where the incidence in men is 2.2 times greater than that in women.^1^ The majority of gastric cancers are adenocarcinomas, which are categorized according to Lauren’s criteria into two main histologically based types of gastric cancer: intestinal-type and diffuse-type gastric carcinomas.^2^ Intestinal-type gastric carcinomas are characterized by the formation of a well-differentiated glandular structure and a relatively well-defined multistep, multifactorial process of histological progression: normal gastric mucosa, chronic atrophic gastritis, intestinal metaplasia, dysplasia and gastric cancer.^2,3^ Intestinal-type gastric carcinomas are commonly attributed to environmental factors and occur primarily in elderly male patients.^4^ Diffuse-type gastric carcinomas are usually not well differentiated, lack adhesion, and spread aggressively throughout the stomach.^2^ These gastric carcinomas are predominantly observed in younger individuals and affect men and women equally.^5–7^ Due to their frequent nodal and distant metastases, they have a worse prognosis than intestinal-type gastric carcinomas.^8,9^ In addition, gastric cancer has been divided into four distinct subtypes by comprehensive molecular classification: genomically stable, chromosomally unstable, microsatellite unstable, and Epstein-Barr virus (EBV)-positive.^10^

*Helicobacter pylori* (*H. pylori*) is a gram-negative, microaerophilic bacterium that was discovered by Marshall and Warren to infect the epithelium of the stomach.^11^ It has been found to have infected roughly half of the world’s population, making it one of the most common human infectious agents worldwide.^12^ While other microorganisms and viruses are unable to survive in the harsh acidic conditions (pH < 2) of the stomach, *H. pylori* colonizes the stomach by penetrating the gastric mucous layer to reach the epithelial cell layer (pH 5–6).^13,14^ Additionally, *H. pylori* can neutralize surrounding acid through the secretion of urease, an enzyme responsible for converting urea into bicarbonate and ammonia.^15^ In this newly acquired niche, *H. pylori* continues to thrive as a monoculture and continuously elicits the host's cellular and humoral immune responses to the site of infection.^16–19^ Consequently, the death of immune and epithelial cells at the site of the immune response provides nutrients to the gastric pathogen, allowing for continued colonization of the stomach over the lifespan of the host.^20^ *H. pylori* infection is transmitted from host to host through the fecal-oral or oral-oral route and is primarily acquired due to poor hygiene and crowded conditions that facilitate transmission of infection mainly among family members.^21^

While the development of gastric cancer is variably influenced by both environmental factors and host genetics, there is undoubtedly a significant impact of *H. pylori* in the development of gastric cancer.^22–24^ In epidemiological studies, *H. pylori* has been identified as an agent of peptic ulcers (gastric ulcers and duodenal ulcers).^25^ Clinico-epidemiological studies have also provided a strong relationship between *H. pylori* infection and the development of mucosa-associated lymphoid tissue (MALT) lymphoma and adenocarcinomas,^26–31^ and the results of subsequent large-scale prospective cohort studies have further supported this association.^32,33^ *H. pylori* infection in Mongolian gerbils has also provided evidence for its role in gastric carcinogenesis.^34–36^ In 1994, the International Agency for Research on Cancer, World Health Organization (IARC/WHO) classified *H. pylori* as a class 1 carcinogen, the only bacterium given this classification.^37^ It is now well accepted that *H. pylori* is the strongest risk factor for the development of both intestinal-type and diffuse-type gastric adenocarcinomas, accounting for ~75% of all human gastric cancer cases.^38,39^

This review summarizes the recent advances in research aimed at the elucidation of the molecular mechanism of gastric carcinogenesis actively driven by the *Helicobacter pylori*-derived CagA oncoprotein, focusing on the CagA-induced pro-oncogenic perturbation of multiple cell signals that coordinately generate functional interplays not only amongst themselves but also with the inflammatory responses of host cells.

## CagA, An *H. Pylori* virulent protein delivered into gastric epithelial cells

*H. pylori* can be divided into two major subpopulations based on the presence or absence of the *cagA* gene that encodes the CagA protein: *cagA*-positive and *cagA*-negative strains.^40^ The *cagA* gene  is one of the 27–31 putative genes that are present in a 40-kilobase genomic DNA segment known as the *cag* pathogenicity island (*cag* PAI).^40,41^ This DNA segment is thought to have been introduced via horizontal transfer from an unknown organism.^40^ Approximately 20 genes found in the *cag* PAI encode components of the type IV secretion system (T4SS), a syringe-like structure that is capable of delivering CagA into the cytoplasm of gastric epithelial cells.^40,41^ CagA is the only effector protein that is known to be secreted by the T4SS.^42–45^ Worldwide, *cagA*-positive strains are responsible for ~60% of the *H. pylori* infections in individuals. Strains isolated in East Asian countries such as Japan, China, and Korea, however, are almost all *cagA*-positive strains.^46^ *cagA*-positive *H. pylori* strains are associated with acute gastritis, peptic ulceration, and gastric cancer.^40,47^ It was first reported in 1995 that infection with *cagA*-positive strains increased the risk of gastric cancer, with a risk that was at least one order of magnitude higher risk than that of *cagA*-negative strains.^48^

*H. pylori* contains several adhesins, including BabA/B, SabA, AlpA/B, HopQ, HopZ, and OipA, that mediate the tight adherence of the bacteria to gastric epithelial cells and consequently initiate and facilitate the formation of the T4SS.^49–51^ The assembled T4SS is composed of three subassemblies, an outer membrane core complex (OMCC), periplasmic ring complex (PRC), and central stalk.^52–54^ The *H. pylori* T4SS is composed of additional components that are not present in the prototypical T4SSs present in other species.^53^ Structural analysis by cryogenic microscopy revealed that the *H. pylori* T4SS contains an expanded OMCC and a symmetry mismatch between the OMCC and the PRC,^55^ and its importance in CagA injection warrants further investigation. Injection of CagA requires recognition by the *H. pylori* T4SS of an Arg-rich CagA-secretion signal sequence (a 20-amino-acid sequence) in the carboxyl-terminal (C-terminal).^56^ Interaction of the C-terminal region of CagA (an 100-amino-acid sequence found near the CagA-secretion signal sequence) with CagF, a secretion chaperone-like protein, of the T4SS is required for injection of CagA.^57,58^ However, the C-terminal region is not sufficient for injection of the CagA protein because both the amino-terminal (N-terminal) region and the C-terminal region are required for this process.^56^ Therefore, the recognition and injection of CagA may be a two-step process requiring first either the C-terminal or N-terminal region followed by the recognition of the second region or a simultaneous recognition of the two distinct regions.

Translocation of CagA into host gastric epithelial cells by *H. pylori* is achieved via a specific interaction between surface adhesins of the bacteria and receptors for the bacterial component on the host cells. In recent studies, carcinoembryonic antigen-related cell adhesion molecules (CEACAMs) have been identified as a set of protein receptors on epithelial cells that are essential for CagA delivery, which is mediated by specific binding of CEACAMs with the outer membrane adhesin HopQ of the bacteria.^50,51,59,60^ The CagL protein, a pilus surface component of the T4SS, has also been reported to be an adhesin that mediates CagA delivery by interacting with and thereby activating ɑ5β1 integrin on target gastric epithelial cells in an arginine-glycine-aspartate (RGD) motif-dependent manner.^61^ CagL stimulates SRC family kinase (SFK) activity, which in turn phosphorylates the delivered CagA.^61^ The ɑ5β1 integrin also interacts with other components of the T4SS, including CagY and CagI, which may cooperatively stabilize the T4SS-host cell interaction.^62^ To further facilitate the delivery of CagA, *H. pylori* induces the membrane phospholipid phosphatidylserine (PS) found in the inner membrane leaflet of epithelial cells to be exposed on the outer membrane.^63^ CagA interacts with the exposed PS to initiate its secretion into the host epithelial cell.^63^

## Molecular structure of the CagA protein

**Fig. 1:**
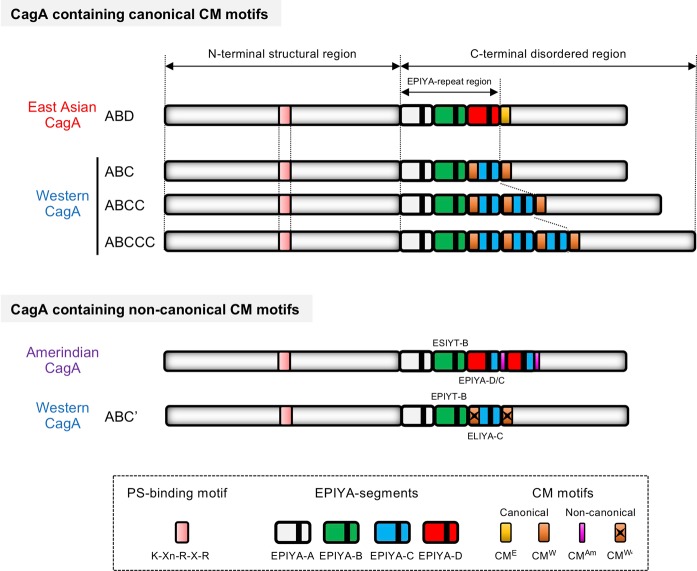
Schematic structure of the *H. pylori* CagA protein. The CagA protein is composed of a structural N-terminal region and an intrinsically disordered C-terminal region. The K-Xn-R-X-R motif is required for CagA to physically associate with the membrane phospholipid phosphatidylserine (PS) in cells. The EPIYA (Glu-Pro-Ile-Tyr-Ala) motifs in the C-terminal region are the tyrosine phosphorylation sites of CagA. The EPIYA-repeat region of CagA includes the common EPIYA segments, EPIYA-A, EPIYA-B, and an East Asian CagA-specific EPIYA-D segment, or a variable number of Western CagA-specific EPIYA-C segments, which are a results of the sequence polymorphisms of the CagA protein. The CM motif, which is composed of 16 amino acid residues, serves as a PAR1b binding site, promoting the multimerization of the CagA protein. Based on sequence polymorphisms, the CM motif is subdivided into 2 groups: a canonical CM motif, which has conserved PAR1b-binding ability, and a noncanonical CM motif, which lacks the binding ability. East Asian CagA possesses a single East Asian CagA-specific CM motif (CM^E^), whereas Western CagA possesses multiple Western CagA-specific CM motifs (CM^W^). The structure of CagA with noncanonical CM motifs, which lack binding ability to PAR1b/MARK2, is shown (bottom panel). The Amerindian CagA, which is derived from the *H. pylori v225* strain, possesses an internally deleted noncanonical CM motif. The ABC’-type Western CagA, which was cloned from the *H. pylori TH2099* strain that had colonized housed macaques, possesses a derivative of the CM^W^ motif with amino acid substitutions (CM^W^’) as well as the atypical EPIYA-C segment that contains the ELIYA sequence.

CagA is a 128–145-kilodalton (kDa) protein that is composed of a structured N-terminal region and an intrinsically disordered/unstructured C-terminal tail.^64–66^ The N-terminus of CagA, which comprises ~70% of the entire CagA, further consists of three structured domains: domain I, domain II, and domain III. Variations in the molecular weight of CagA are due to the structural polymorphisms in its C-terminal region, which exist in distinct strains of *H. pylori*^64^ (Fig. 1). Once injected into the host gastric epithelial cells, CagA is localized to the inner leaflet of the plasma membrane. The mechanism for intracellular localization of CagA is strongly dependent on the status of apicobasal polarity of the epithelial cells in which CagA is delivered. In polarized epithelial cells, the positively charged phosphatidylserine (PS)-binding K-Xn-R-X-R motif located in domain II of the N-terminal region of CagA interacts with PS, which plays an important role in tethering CagA to the membrane^63^ (Fig. 1). However, in nonpolarized epithelial cells, the intrinsically disordered C-terminal tail plays an essential role in the localization of CagA to the membrane.^67^

While the bacterial CagA protein has no significant homology in its primary structure with known proteins in humans and other species, it undergoes tyrosine phosphorylation by host tyrosine kinases specifically on the Glu-Pro-Ile-Tyr-Ala (EPIYA) motifs in the C-terminal polymorphic region (EPIYA-repeat region)^45,68,69^ (Fig. 1). From the sequence flanking the EPIYA motif, 4 distinct EPIYA segments have been identified in the CagA protein: EPIYA-A, EPIYA-B, EPIYA-C, and EPIYA-D.^70,71^ *H. pylori* strains in regions excluding East Asian countries contain the CagA protein with EPIYA segments arranged as EPIYA-A (32 amino acids), EPIYA-B (40 amino acids), and EPIYA-C (34 amino acids) and is thereby referred to as Western CagA or ABC-type CagA.^72^ The EPIYA-C segment is present in variable numbers of copies among distinct Western CagA variants, typically represented in tandem between 1 to 3 times.^72^ The EPIYA-repeat region of CagA found in East Asian countries also possesses EPIYA-A and EPIYA-B segments but, instead of the tandem EPIYA-C segment, contains a distinct EPIYA-containing segment termed EPIYA-D (47 amino acids), and the CagA protein is referred to as East Asian CagA or ABD-type CagA^70^ (Fig. 1).

Due to the variation of the sequence flanking the tyrosine (Y) residue, the distinct EPIYA segments are tyrosine-phosphorylated selectively by different kinases. SFKs, including c-Src, Fyn, Lyn, and YES, specifically phosphorylate the EPIYA-C and EPIYA-D segments, while the c-Abl tyrosine kinase phosphorylates all of the segments.^73–75^ Interestingly, CagA delivered into host cells has been found to only be phosphorylated on 1 or 2 EPIYA segments, but never 3 or more, in experiments utilizing *H. pylori* strains with ABC-type CagA.^75^ EPIYA-A and EPIYA-C or EPIYA-B and EPIYA-D are preferably phosphorylated in combination in Western CagA and East Asian CagA, respectively.^75^ Therefore, there may be a stepwise event in which EPIYA-C or EPIYA-D is phosphorylated by SFKs at the start of an infection followed by phosphorylation of EPIYA-A or EPIYA-B by c-Abl at a subsequent time point.^75^ Dephosphorylation of the given SRC/c-Abl-mediated phosphotyrosyl residues of the EPIYA motifs is mediated by SH2 domain-containing protein tyrosine phosphatase (PTPase) 1 (SHP1) in cells.^76^ Since the binding/scaffolding capability of EPIYA motifs to intracellular proteins is tyrosine phosphorylation-dependent, the phosphorylation status of EPIYA motifs strongly influences the pathobiological activity of the CagA protein.

At the plasma membrane, the delivered CagA is present as a multimerized complex, which requires the conserved 16-amino-acid sequence [CagA-multimerization (CM) motif] in the C-terminal region of CagA.^77,78^ The CM motif is located immediately distal to either the EPIYA-C segment in Western CagA or the EPIYA-D segment in East Asian CagA^77^ (Fig. 1). There are alterations in 5 amino acid residues in the CM motif between Western CagA and East Asian CagA. The Western CagA CM motif is therefore termed CM^W^, and East Asian CagA is termed CM^E^.^79^ The 16 amino acids at the N-terminal sequence of EPIYA-C are identical to those in CM^W^, and therefore, CagA containing multiple EPIYA-C segments also contains increased numbers of CM motifs^77^ (Fig. 1). In addition to CagA multimerization, the CM motif is also involved in the stability of delivered CagA, the degradation of which is mediated by an autophagy-dependent mechanism.^80–83^

## Pro-oncogenic scaffolding functions of CagA that perturb host cell signaling

### *Deregulation of SHP2, the pro-oncogenic PTPase involved in the regulation of cell growth, motility, and morphology*

When the CagA protein is expressed in human gastric epithelial cells (*e.g*., the AGS cell line), the hummingbird phenotype, a morphological change characterized by an elongated cell shape with elevated cell motility, is observed in a CagA-tyrosine-phosphorylation-dependent manner.^84,85^ Since the unique morphology of the hummingbird cell is similar to that of cells treated with hepatocyte growth factor (HGF),^84,86^ it was suggested that the tyrosine-phosphorylated CagA is capable of aberrantly emitting a mitogenic cue, which is reminiscent of that generated upon HGF stimulation. Posttranslational tyrosine phosphorylation of proteins is known to function as a switch that mediates intracellular signal transduction to an SH2 domain-containing protein through a phospho-tyrosine/SH2-domain interaction, which is utilized in mitogenic signaling pathways triggered by growth factors.^86^

**Fig. 2:**
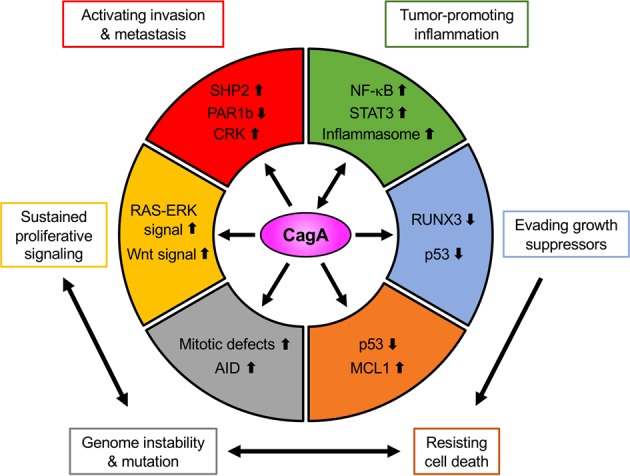
Schematic representation of the pro-oncogenic actions of the *H. pylori* CagA oncoprotein. When delivered into gastric epithelial cells, the CagA oncoprotein perturbs multiple intracellular signaling pathways and then promotes malignant transformation of the host cells by providing cancer-hallmark capabilities. There is a mutual feedforward stimulatory mechanism between the pro-oncogenic activities of CagA and pro-inflammatory responses.

SH2 domain-containing PTPase 2 (SHP2) was the first identified SH2 domain-containing protein that could bind to the tyrosine-phosphorylated EPIYA-C (EPIpYA-C) or EPIYA-D (EPIpYA-D) site of the CagA protein.^85^ SHP2 is a nonreceptor type PTPase encoded by the *PTPN11* gene that possesses two regulatory SH2 domains, an enzymatic PTPase domain and a C-terminal tail region. In physiological conditions, SHP2 is in an enzymatically inactive conformation, in which catalytic reaction to its substrates is interfered with due to the autoinhibitory intramolecular interaction of its SH2 domains with the PTPase domain. Binding of a tyrosine-phosphorylated peptidyl ligand to the SH2 domains changes the structure of the PTPase from the autoinhibitory/closed conformation to an enzymatically active/opened conformation.^87^ Genetic analysis has provided evidence that cytoplasmic SHP2 is activated by growth factor stimuli and that SHP2 activity is required for full activation of the RAS-RAF-MEK-ERK pathway, a pro-oncogenic signaling pathway involved in the proliferation and differentiation of cells (*e.g*., neuroblastoma cells).^88,89^ The binding of EPIpYA-C or EPIpYA-D of CagA protein to SHP2 aberrantly maintains the active/opened conformation of the PTPase, thereby constitutively activating the downstream MEK-ERK kinases^90^ (Fig. 2). SHP2 has also been reported to regulate the signaling pathways associated with cell morphology and motility.^88^ For instance, SHP2 dephosphorylates and inactivates focal adhesion kinase (FAK), a key regulator of the dynamics of focal adhesions.^91^ Therefore, the tyrosine-phosphorylated CagA-induced hummingbird phenotype is due to the perturbation of both SHP2-MEK-ERK signaling and SHP2-FAK signaling through EPIpYA-C-mediated or EPIpYA-D-mediated aberrant activation of the SHP2 phosphatase^70,85,91,92^ (Fig. 2).

Since gain-of-function mutations of the *PTPN11* gene, which encodes SHP2, have been found in patients with hematopoietic malignancies [juvenile myelomonocytic leukemia (JMML), childhood myelodysplastic syndrome, B-cell acute lymphoblastic leukemia and acute myelocytic leukemia] and solid tumors, SHP2 is thought to be a *bona fide* oncoprotein.^93–95^ In addition to the SHP2 substrates associated with RAS-ERK signaling and FAK, Parafibromin [also known as cell division cycle 73 (CDC73)], a core component of the RNA polymerase II-associated factor (PAF) complex, which is localized in the nucleus of cells, has also been reported as a substrate of SHP2.^96^ Parafibromin is a transcriptional scaffold protein that promotes transcription of target genes transactivated by transcriptional coactivators/effectors, such as β-catenin, Gli1, NICD (Notch intracellular domain) and YAP/TAZ, through the formation of complexes dependent on the status of tyrosine phosphorylation.^96–99^ Regarding the SHP2-parafibromin axis, an immunohistochemical study of *H. pylori*-infected gastric epithelia has shown that a small fraction of CagA is present in the nucleus.^100^ Although further investigation is required, Parafibromin and its downstream transcriptional circuit could be a potential target of the CagA/SHP2 complex in the nucleus.

Unlike EPIpYA-C and EPIpYA-D, the EPIpYA-A and EPIpYA-B motifs are utilized as docking sites for another SH2-containing protein, C-terminal SRC kinase (CSK). CSK is a protein tyrosine kinase that is responsible for the phosphorylation of an inhibitory phosphorylation site of SFKs, which phosphorylate EPIYA-C and EPIYA-D of CagA.^101^ Therefore, CagA/CSK complex formation contributes to the attenuation of CagA-SHP2 signaling by downregulating phosphorylation on EPIYA-C and EPIYA-D via inhibition of SFKs.^102^ In addition to SHP2 and CSK, proteomic screening of intracellular host proteins that physically associate with tyrosine-phosphorylated CagA peptides has revealed five additional SH2-domain-containing proteins as potential CagA-binding partners (SHP1, PI3K, GRB2, GRB7 and RAS-GAP), indicating that the pathobiological roles of CagA in hijacking multiple signaling pathways in the host cells  are also mediated through these tyrosine-phosphorylated proteins.^103^

### *Inhibition of PAR1b, the serine/threonine kinase that regulates cell polarity and microtubule dynamics*

Tyrosine phosphorylation of the EPIYA motifs is not necessary for all of the pathobiological actions of the CagA protein. Proteomic analysis of the coprecipitates of the CagA protein revealed that partitioning defective-1 (PAR1) family serine/threonine kinases interact with CagA, irrespective of the tyrosine phosphorylation status of the CagA protein.^104^ The CagA-PAR1 interaction requires the CM motif of CagA, which directly binds to the catalytic cleft of the PAR1 kinase, thereby inhibiting its kinase activity.^104,105^ Due to the high sequence homology in the kinase domain among the isoforms, CagA is capable of inhibiting all four mammalian PAR1 isoforms, including PAR1b, a predominant isoform in epithelial cells.^79,106^ In polarized epithelial cells, PAR1b is localized to the basolateral membrane domain, where it is distinguished from that in the apical membrane domain by the tight junctions present between them. Along with the other PAR proteins, PAR1b plays an indispensable role in maintaining tight junctions, which control paracellular permeability across the epithelial cell monolayer, a fundamental role in the establishment and maintenance of apicobasal polarity in epithelial cells.^107^ CagA expression in a polarized epithelial cell monolayer (*e.g*., MDCK cells) causes polarity defects characterized by nonpolarized distribution of tight junctional proteins and basolateral proteins, such as ZO-1 and E-cadherin, respectively, which impairs tight junctional barriers of the monolayer via the CM motif-dependent inhibition of PAR1b (Fig. 2).^104^ Additionally, PAR1 kinases have an alternative name, MARKs (microtubule affinity-regulating kinases), and these were independently cloned and identified as kinases crucial for maintaining microtubule dynamics via the phosphorylation of microtubule-associated substrates.^108^ Furthermore, it was found that CagA expression in mitotic gastric epithelial cells leads to a delay in the prophase/metaphase transition, which is concomitantly associated with spindle misorientation at the onset of anaphase, followed by chromosomal segregation with an abnormal division axis. The effects of CagA in cell division are abolished by the coexpression of PAR1b, indicating that CagA-mediated inhibition of PAR1b is required for the prophase/metaphase delay and the subsequent spindle misorientation, those of which may be caused by perturbed microtubule stability as well as microtubule-based spindle dysfunction^109^ (Fig. 2). In addition, PAR1b is known to phosphorylate the RhoA-specific guanine exchange factor H1 (GEF-H1) to inhibit GEF activity, which is required for the induction of RhoA-dependent stress fiber formation.^110^ Therefore, CagA alters not only the microtubule-based cytoskeletal system but also the actin-based cytoskeletal system, the coordination of which may contribute to the induction of abnormal cell polarity, cell morphology and cell movement in CagA-expressing cells (Fig. 2). Moreover, it has recently been reported that MST1/2 (mammalian Ste20-like kinase 1/2), a component of the tumor-suppressing Hippo signaling pathway, is a novel substrate of PAR1b.^111^ Therefore, further investigation may reveal the involvement of perturbed MST1/2-LATS1/2-YAP/TAZ signaling induced by the CagA-PAR1b complex in the pathobiological action of the CagA protein.

### *Adherence junctional defects and Wnt activation, which induce aberrant cell-fate reprograming*

In addition to the tight junctional defects, CagA has been reported to target an adherence junctional component, which is functionally associated with Wnt signaling as well as epithelial-mesenchymal transition (EMT) (Fig. 2). CagA has the ability to physically interact with the cytoplasmic domain of E-cadherin in a CM motif-dependent manner. The CagA/E-cadherin interaction impairs and destabilizes E-cadherin/β-catenin complex formation and aberrantly translocates the membranous fraction of β-catenin to the nucleus, where it subsequently transactivates Wnt target genes in a β-catenin/TCF-dependent manner.^112,113^ The following alternative mechanisms of CagA-mediated Wnt activation have been reported: CagA potentiates Wnt signaling through activation of phosphoatidylinositol-3-kinase (PI3K)-AKT signaling by physically interacting with the HGF-stimulated c-Met receptor via the CM motif in a tyrosine phosphorylation-independent manner;^114^ CagA physically associates with glycogen synthase kinase (GSK)-3β via its C-terminal region, thereby sequestering GSK-3β and subsequently potentiating Wnt activation;^115^ and CagA promotes the proteasome-mediated degradation of the RUNX3 tumor suppressor, which attenuates β-catenin/TCF-dependent transcription.^116,117^ It has been reported that the aberrant activation of Wnt signaling by CagA induces ectopic expression of intestine-specific caudal-related homeobox transcription factors (CDX1 and CDX2), and this transcriptionally activates the stemness-associated reprograming factors Sal-like protein 4 (SALL4) and Krüppel-like factor 5 (KLF5).^118,119^ These findings suggest that ectopic expression of these transdifferentiation-associated transcriptional factors by CagA in gastric epithelial cells may play a key role in the development of intestinal metaplasia. Regarding the pathobiological action of CagA in adherens junctions, it has been reported that tyrosine-phosphorylated CagA has the ability to disrupt adherens junctions by forming a complex with CRK adaptor proteins (CRK-I, CRK-II and CRK-L)^120^ (Fig. 2). Impairment of the adherens junctions and tight junctions in polarized epithelial cells by CagA is an important cue that triggers EMT-like dedifferentiation, which is characterized by morphological change, invasive motility, and induction of mesenchymal-specific proteins such as snail.^121–123^

### *Induction of genomic/chromosomal instability concomitant with inactivation of tumor suppressor p53*

CagA was originally identified as a product of the “*cytotoxin-associated gene A (cagA)*”. However, the CagA protein itself does not show cytotoxic activity against host epithelial cells. Along with the degradation of the tumor suppressor RUNX3, CagA subverts the tumor suppressor function of apoptosis-stimulating protein of p53 2 (ASPP2), also known as Bcl2-binding protein (Bbp)/tumor suppressor p53-binding protein 2 (p53BP2), by physical interacting with the N-terminal region of CagA (Fig. 2). Through the interaction with ASPP2, CagA counteracts the p53-mediated apoptotic response upon cellular stress by promoting the degradation of the p53 tumor suppressor.^124–126^ CagA has also been reported to enhance p53 degradation by activating E3 ubiquitin ligases, HDM2 (human double minute 2) and ARF-BP1 (ARF-binding protein 1), both of which are negatively regulated by p14ARF.^127,128^ A recent study revealed that CagA aberrantly promoted the induction of TRIP12 (thyroid hormone receptor-interacting protein 12), an E3 ubiquitin ligase for the p14ARF protein.^129^ Therefore, loss of the *ARF* gene locus as well as the CagA-mediated degradation of the p14ARF protein in gastric epithelial cells may potentiate the p53 degradation induced by CagA.

In addition to stimulating proteasomal degradation of p53, CagA has been reported to promote the acquisition of spontaneous loss-of-function mutations in the *p53* gene. Chronic infection with *cagA*-positive *H. pylori* in the gastric epithelium induces ectopic expression of AID (activation-induced cytidine deaminase), a DNA/RNA editing enzyme essential for the somatic hypermutation and class-switch recombination of immunoglobulin genes, via nuclear factor-κB (NF-κB) activation in a T4SS-dependent manner. The ectopic expression of AID results in the accumulation of nucleotide alterations, including spontaneous loss-of-function mutations in the *TP53* tumor suppressor gene^130^ (Fig. 2). Furthermore, chronic exposure to the CagA protein induces aneuploidy/polyploidy in gastric epithelial cells, which may be due to the CagA/PAR1b-mediated defects in microtubule-based mitotic spindle formation^109^ (Fig. 2). Although the molecular mechanism is still under investigation, it was recently reported that CagA-mediated PAR1b inhibition induced DNA double-strand breaks (DSBs).^131^ Consistently, infection of gastric epithelial cells with *cag* PAI-positive *H. pylori* strains has been shown to induce DSBs, which are concomitantly associated with reduced expression of the DSB repair factor *RAD51*.^132^ Notably, *H. pylori*-induced gastritis, particularly that induced by *H. pylori cagA*-positive strains, is associated with CpG hypermethylation of *MGMT*, the gene encoding the DNA repair protein O6-methylguanine DNA methyltransferase (MGMT).^133^ CagA also upregulates expression of the anti-apoptotic protein MCL1 (myeloid cell leukemia sequence-1) through activation of pro-survival MEK-ERK signaling^134^ (Fig. 2). Collectively, these observations indicate that upon delivery of CagA, host gastric epithelial cells not only acquire resistance to cell death via inactivation of the p53 tumor suppressor but also induce chromosomal/genomic instability, both of which are hallmarks of cancer cells^135^ (Fig. 2).

## Sequence Polymorphisms of CagA that Confer Differential Oncogenic Potential

**Fig. 3:**
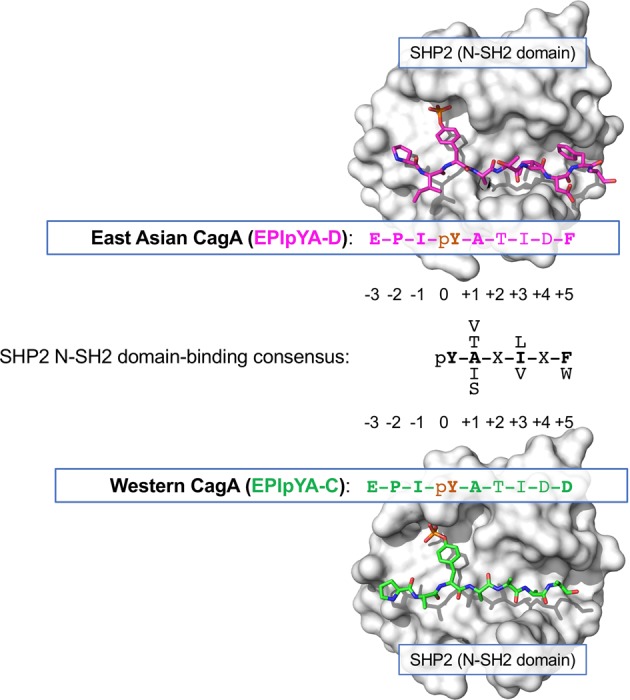
Polymorphism-dependent differential binding of the EPIYA motif of CagA to SHP2. The consensus sequence of the phosphotyrosyl peptide for binding to the N-SH2 domain of SHP2 is shown. The sequence of the amino acid residues following the EPIpYA-D motif in East Asian CagA (magenta) is perfectly matched to the N-SH2 domain-binding consensus sequence (black). In contrast, a single mismatched amino acid residue is shown at the pY + 5 position in the EPIpYA-C peptide derived from Western CagA (light green). The EPIpYA/N-SH2 physical interactions are visualized by the crystal structures of the SHP2 tandem SH2 domains (surface representation, white) complexed with the EPIpYA-D peptide (magenta) and EPIpYA-C peptide (light green). The phospho-tyrosine residues (pY) in the EPIYA peptides are observed to interface with a deep pocket of the SH2 groove. The phenylalanine residue (F) at the pY + 5 position of the EPIpYA-D peptide contributes to a binding interface with high affinity for the SH2 domain.

Since the CagA protein has an *H. pylori* strain-specific polymorphism in its primary structure, especially in its C-terminal region, which contains distinct EPIYA-A/B/C/D segments and CM motifs, the pathobiological activity of CagA differs among the distinct CagA species. The amino acid sequence flanking the phospho-tyrosine (pY) residue of the EPIYA-D segment is perfectly matched to the consensus motif of the phospho-tyrosyl peptide that can bind to the SH2 domain of SHP2, while a single amino acid mismatch is present at the pY + 5 position in the pY-flanking sequence of the EPIYA-C segment^70,71^ (Fig. 3). A quantitative biochemical study revealed that the East Asian CagA (ABD)-specific EPIpYA-D peptide binds to the N-SH2 domain of SHP2 ~100-fold more strongly than the Western CagA (ABC)-specific EPIpYA-C peptide. The phenylalanine (F) residue at the pY + 5 position of the EPIpYA-D peptide was found to form a high-affinity monovalent bond with the hollow pocket on the N-SH2 phosphopeptide-binding floor of SHP2^136^ (Fig. 3). Accordingly, East Asian CagA exhibits a stronger ability to bind/deregulate SHP2 and a greater capability to induce SHP2-dependent morphological changes in gastric epithelial cells than Western CagA.^70,136^ Collectively, the findings reveal that the East Asian CagA-specific EPIpYA-D motif is qualitatively very different from the Western CagA-specific EPIpYA-C motif in terms of the biological activity required for deregulation of the SHP2 oncoprotein, which may causatively account for the higher incidence of gastric cancers in East Asian countries than in Western countries. Notably, clinical evidence supports that gastric cancer is more closely associated with *H. pylori* strains carrying East Asian CagA than with *H. pylori* strains carrying Western CagA in geographical regions where two distinct strains cocirculate.^137–139^

In contrast to the East Asian CagA species, the great majority of which is composed of CagA with a single EPIYA-D segment for SHP2-binding, Western CagA species can be subdivided into two major groups: CagA with a single EPIYA-C segment for SHP2-binding (type I Western CagA) and CagA with multiple EPIYA-C segments (type II Western CagA), which account for ~60–70% and 30–40% of all Western CagA species, respectively.^140,141^ Type II Western CagA is characterized by tandemly arranged EPIYA-C duplication or triplication, which is thought to be causally due to genetic recombination that occurred in the EPIYA-repeat region^141,142^ (Fig. 1). Biochemical analysis revealed that type II Western CagA exhibits stronger binding to SHP2 than does type I Western CagA. Moreover, even among the type II Western CagA species, a CagA with a larger number of EPIYA-C segments displays stronger binding to SHP2 than a CagA with a smaller number of EPIYA-C segments.^140,143,144^ Based on the fact that the two SH2 domains (N-SH2 and C-SH2) are present in a single SHP2 molecule, type II Western CagA is thought to acquire the ability to bind to SHP2 with high affinity via divalent interaction of the multiplicated EPIpYA-C segments with N-SH2 and C-SH2 domains. In contrast, type I Western CagA is thought to bind to SHP2 via the monovalent low-affinity interaction of EPIpYA-C with N-SH2 or C-SH2.^136^ These quantitative differences in the SHP2-binding ability between the two types of Western CagA account for the differential degrees of SHP2-dependent pathobiological actions of type I and type II Western CagA in gastric epithelial cells, since the levels of the induced hummingbird and invasive phenotypes are positively correlated with the number of EPIYA-C segments in distinct Western CagA types.^70,140,145^ Consistently, another polymorphism in the EPIYA repeat region of CagA that confers multiplication of EPIYA-A and EPIYA-B segments also proportionally attenuates the CagA/SHP2-dependent induction of the hummingbird phenotype through EPIpYA-A- or EPIpYA-B-mediated activation of CSK, which in turn inhibits SRC-dependent tyrosine phosphorylation of CagA on EPIYA-C or EPIYA-D motifs.^143^ Notably, several clinico-epidemiological studies have shown that infection with *H. pylori* strains carrying type II Western CagA is strongly associated with the risk of gastric cancer when compared to infection with *H. pylori* carrying type I Western CagA.^146,147^ This further suggests that the quantitative difference in SHP2 deregulation is a crucial factor that determines the distinct level of oncogenic activity of Western CagA species.

Comprehensive analysis of sequence polymorphisms in EPIYA motifs of the CagA protein showed that the EPIYA-B site was the most polymorphic among the 4 distinct EPIYA-A/B/C/D sites. While almost all (>98%) of the EPIYA-C sites are composed of the authentic EPIYA sequence, the authentic EPIYA-B sequence accounts for only 55.5% of the entire population of EPIYA-B polymorphisms and is also shared by the EPIYT-B sequence (32.9%) and others. CagA physically interacts with the SH2 domain of PI3K via the tyrosine-phosphorylated EPIYA-B site, which thereby potentiates PI3K-AKT signaling. A coimmunoprecipitation experiment and structural modeling of the EPIpYA-B or EPIpYT-B peptide bound to the SH2 domain of PI3K indicated that the threonine (T) residue at the pY + 1 position of EPIpYT-B gives higher affinity to the SH2 domain for binding compared to that of the authentic EPIpYA-B sequence.^148^ Since clinical evidence shows a weaker association of CagA carrying the EPIYT-B polymorphism with gastric cancer than CagA carrying the EPIYA-B motif, involvement of the EPIYA-B polymorphism-dependent differential activation of PI3K-AKT requires further investigation.^148^

*H. pylori* strain-specific polymorphisms of the CagA protein are also observed in the CM motif. Among CagA species isolated so far, the CM motif can be classified into two groups according to the PAR1b-binding capability: a canonical CM motif, which conserves the binding ability, and a noncanonical CM motif, which does not. The canonical CM motif is further subdivided into two groups, the Western CagA-specific CM^W^ motif and the East Asian CagA-specific CM^E^ motif. Most of the Western CagA species possess 2-4 repeated CM^W^ motifs, with some exceptions. On the other hand, East Asian CagA species possess a single CM^E^ motif^77,79,149^ (Fig. 1). In a similar fashion to the case of the EPIYA-C/D polymorphism, the single CM^E^ motif exhibits approximately 2-fold stronger PAR1b-binding ability than a single CM^W^ motif. An increased number of CM^W^ motifs in a single Western CagA protein exhibits an enhanced PAR1b-binding ability in a synergistic manner. The differential PAR1b-binding activity among the distinct CagA species is proportional to the degree of pathobiological activity of the CagA/PAR1b complex such as tight junctional disruption and stress fiber formation in gastric epithelial cells.^150^ The biological activities of CagA possessing the noncanonical CM motif have also been investigated. The CagA species with an Amerindian-type CM motif (CM^Am^ motif), which is thought to be an internally deleted derivative of the canonical CM motif, showed neither PAR1b-binding nor alterations in epithelial cell polarity, while EPIpYA-mediated SHP2-binding was retained^150,151^ (Fig. 1). Related to this, it has been reported that the *cagA* gene isolated from an *H. pylori* strain that colonized housed macaque encodes an ABC-type Western CagA derivative (ABC’-type CagA), which is characterized by variations in residues in both the EPIYA-C segment and the CM motifs (Fig. 1). Due to the amino acid substitutions present in the noncanonical CM motifs, the ABC’-type CagA lacks binding ability to PAR1b. Additionally, the atypical EPIYA-C segment, which is characterized by amino acid substitutions in the EPIYA-C motif (ELIYA), causes impaired SHP2-binding activity of ABC’-type CagA. In contrast to the standard *H. pylori* strain with ABC-type *cagA*, infection of a gastric organoid with *H. pylori* carrying the ABC’-type *cagA* failed to elicit CagA-dependent epithelial disruption, suggesting that binding to SHP2 and PAR1b may play a key role in the virulence of distinct CagA species.^152^

## Oncogenic potential of CagA in vivo

The pathobiological actions of CagA observed in gastric epithelial cells in vitro suggest that CagA perturbs multiple signaling pathways highly associated with carcinogenesis. Consistently, a number of epidemiological studies have shown an intimate association of CagA with the development of *H. pylori*-associated gastric cancer.^153–155^ However, at that time, no in vivo evidence convincingly supported a crucial role of CagA in gastric carcinogenesis, although a key role of *cag* PAI, encoding the T4SS for CagA delivery, was indicated.^36,156^ To clarify the causal link between CagA and in vivo oncogenesis, transgenic mice that express wild-type CagA protein (East Asian CagA) ubiquitously throughout the body were generated (*WT cagA*^*E*^-Tg mice).^157^ Transgenic expression of the CagA protein caused hyperplasia of the gastric epithelium. Most importantly, in some of the *WT cagA*^*E*^-Tg mice, adenocarcinoma spontaneously developed in the stomach and small intestine. Since the CagA protein produced in the transgenic system indeed underwent tyrosine phosphorylation on EPIYA motifs in cells, abnormal signal transduction perturbed by tyrosine-phosphorylated CagA was thought to be involved in the in vivo oncogenicity of CagA. To investigate the role of the tyrosine phosphorylation of CagA, *cagA* transgenic mice that expressed tyrosine phosphorylation-resistant CagA (PR CagA), a CagA derivative with tyrosine-to-phenylalanine substitutions in all EPIYA-A/B/D motifs, were also established (*PR cagA*^*E*^-Tg mice). In contrast to the *WT cagA*^*E*^-Tg mice, the *PR cagA*^*E*^-Tg mice did not show gastrointestinal abnormalities, suggesting the requirement of tyrosine phosphorylation on EPIYA motifs for the in vivo pathogenicity of CagA protein in the gastrointestinal tract in mice.^157^ In addition, the *WT cagA*^*E*^-Tg mice showed leukocytosis associated with hypersensitivity to hematopoietic cytokines, including interleukin-3 (IL-3) and granulocyte-macrophage colony-stimulating factor (GM-CSF), in bone marrow cells. Some of those *WT cagA*^*E*^-Tg mice developed myeloid leukemias as well as B-cell lymphomas.^157^ The development of such hematological abnormalities, including hematological malignancies, occurred in mice expressing a gain-of-function mutant of SHP2.^158,159^ In contrast, the *PR cagA*^*E*^-Tg mice, which systemically expressed a CagA protein lacking SHP2-binding ability, did not show any hematological abnormalities, even though the expression levels of PR CagA in *PR cagA*^*E*^-Tg mice were comparable to or even higher than those of wild-type CagA in *WT cagA*^*E*^-Tg mice, suggesting an indispensable role of the tyrosine phosphorylation on EPIYA motifs in the hematological diseases observed in *WT cagA*^*E*^-Tg mice.^157^ The involvement of SHP2 deregulation by CagA was supported by further in vivo evidence obtained from transgenic mice systemically expressing wild-type Western CagA (*WT cagA*^*W*^-Tg mice) at levels comparable to those of East Asian CagA in *WT cagA*^E^-Tg mice. The *WT cagA*^W^-Tg mice developed gastric epithelial hypertrophy and gastrointestinal tumors in addition to lymphoid malignancies but did not develop myeloid abnormalities, which were found in *WT cagA*^*E*^-Tg mice. Furthermore, the incidence of tumors in *WT cagA*^*W*^-Tg mice was significantly lower than that in *WT cagA*^*E*^-Tg mice, indicating that Western CagA is qualitatively less oncogenic than East Asian CagA in vivo.^160^

Another *cagA*-transgenic (*cagA*-Tg) animal with either systemic CagA expression or tissue-specific CagA expression in the distal esophagus and anterior intestine was generated using zebrafish. The *cagA*-Tg zebrafish showed hyperplasia in the adult intestinal epithelium. Moreover, transgenic expression of the *cagA* gene in *p53*-null zebrafish induced intestinal small-cell carcinoma and adenocarcinoma.^161^ These results collectively suggest a key role of tyrosine phosphorylation in the in vivo pathobiological/oncogenic activity of the CagA protein, which may cooperate with alterations of cancer-associated genes in host cells.

Although *cagA*-positive *H. pylori*-associated human gastric cancers frequently coexist with precancerous chronic atrophic gastritis,^162^ the gastrointestinal neoplasms that developed in the *WT cagA*-Tg mice, as well as the hyperplasia shown in the *cagA*-Tg zebrafish did not show any overt inflammation.^157,160,161^ These observations are consistent with the notion that the CagA protein *per se* is not a potent inflammogen^163–165^ and indicate that inflammation is not necessarily required for the oncogenesis evoked by CagA in vivo. However, chronic inflammation, which is associated with *cagA*-positive *H. pylori* infection, has been reported to promote the oncogenesis of cancer-predisposed gastric epithelial cells.^165^ To experimentally investigate whether the inflammatory response can enhance the pro-oncogenic action of CagA in vivo, *WT cagA*^E^-Tg mice, which systemically expressed CagA, were treated with a colitis inducer, dextran sulfate sodium (DSS). The DSS-treated *WT cagA*^E^-Tg mice showed a higher incidence of colonic dysplasia than did non-DSS-treated *WT cagA*^E^-Tg mice and DSS-treated control mice, suggesting that colonic inflammation accelerated the pro-oncogenic potential of CagA in this experimental model in the colon.^166^

## Induction of proinflammatory host responses by *cagA*-Positive *H. pylori*

Clinico-pathological observations strongly indicated proinflammatory actions of the CagA protein in gastric pathogenesis.^167^ Animal infection studies showed that *cagA*-positive *H. pylori* strains elicited a greater severity of mucosal inflammation than *cagA*-negative strains.^137,156^ DSS-treated *WT cagA*^E^-Tg mice, which systemically expressed CagA, also exhibited deterioration of DSS-induced colitis compared to DSS-treated wild-type control mice.^166^ Immunoblot analysis of the colon revealed a substantial decrease in the level of IκB (inhibitor of NF-κB), which prevented nuclear translocation of NF-κB, in a CagA/PAR1b-dependent manner.^166^ Although the CagA-mediated IκB reduction *per se* was insufficient to activate NF-κB signaling, a primary mediator of the inflammatory response, it made cells hypersensitive to stimuli that activate NF-κB.^166,168^ It was reported that CM motif-dependent NF-κB activation by CagA is also induced by activation of IKK (IκB kinase), which promotes IκB degradation, through the CagA-c-Met-PI3K-AKT axis.^114^ IKK activation by CagA was also reported to be mediated by the activation of TGF-β-activated kinase 1(TAK1). CagA physically interacts with TNF receptor-associated factor 6 (TRAF6)-TAK1 complex and promotes aberrant poly-ubiquitination/activation of TAK1, leading to activation of IKK.^169^ Independent of NF-κB signaling, CagA induces the proinflammatory cytokine interleukin-1β (IL-1β), which is cleaved/matured by the inflammasome complex in epithelial cells^166^ (Fig. 2). It has also been reported that CagA gives rise to the induction of the pro-inflammatory cytokine interleukin-8 (IL-8), probably due to EPIpYA-B-dependent PI3K activation, which may be influenced by polymorphisms at the EPIYA-B site.^148^ In addition, CagA is reported to activate STAT3 (signal transducer and activator of transcription 3), which is a proinflammatory transcription factor, via the IL-6/gp130 signaling pathway by a mechanism independent of the tyrosine phosphorylation of CagA^170^ (Fig. 2).

*H. pylori*-evoked proinflammatory responses of host cells are caused not only by CagA but also by other factors of the bacterium. It has been reported that bacterial peptidoglycan delivered through the T4SS from *H. pylori* stimulates Nod1 (nucleotide-binding oligomerization domain 1), a cytoplasmic pathogen recognition receptor, in epithelial cells and activates NF-κB, which induces inflammatory cytokines including IL-1, IL-6, IL-8, TNFα (tumor necrosis factor α) and/or IFN-γ (interferon-γ).^171,172^ The involvement of *H. pylori* lipopolysaccharide (LPS) in NF-κB activation in gastric epithelial cells has been reported; *H. pylori* LPS itself may stimulate NF-κB through TLR2 (toll-like receptor 2) or TLR4-mediated signaling.^173^ However, recent studies have revealed that T4SS-delivered ADP-*glycero*-β-D-*manno*-heptose (ADP heptose), an LPS metabolite that is a novel pathogen-associated molecular pattern (PAMP) of gram-negative bacteria, including *H. pylori*, plays a central role in NF-κB activation by *H. pylori* via ALPK1 (α-kinase 1)-TIFA (TRAF-interacting protein with forkhead-associated domain) signaling.^174–176^ In addition to the *H. pylori*-epithelial cell interaction, *H. pylori* also infects immune cells in the gastric epithelial niche and thereby activates the NLRP3 inflammasome to induce IL-1β in a *cag* PAI-dependent manner.^177–179^ It is thus thought that *cagA*-positive *H. pylori* induces a host inflammatory response, which enhances the oncogenic potential of CagA, by perturbing immune signaling in both gastric epithelial cells and infiltrating immune cells.

## Gastric carcinogenesis evoked by the *H. Pylori* CagA oncoprotein

It is generally thought that normal cells evolve progressively to a neoplastic state by successively acquiring the following ten hallmark capabilities: sustained proliferative signaling, evasion of growth suppressors, activation of invasion/metastasis, replicative immortality, induction of angiogenesis, resistance to cell death, genome instability/mutability, induction of tumor-promoting inflammation, evasion of immune destruction, and deregulation of cellular energetics.^135^ Based on this concept, the multistep process of human tumor pathogenesis can be accounted for by the stepwise acquisition of these characteristics, which subsequently enable normal cells to become tumorigenic and malignant.^135^ *cagA*-positive *H. pylori*-associated gastric carcinogenesis is also thought to progress in this stepwise fashion.^2,3^ *In vitro* and in vivo analyses of the CagA protein thus far have collectively characterized CagA as a *bona fide* oncoprotein that confers six of the ten cancer hallmark capabilities to host cells. Five of these hallmarks are induced cell-autonomously: (1) sustained proliferative signaling (SHP2-ERK and Wnt signaling), (2) evasion of growth suppressors (inactivation of RUNX3 and p53), (3) activation of invasion and metastasis (hummingbird phenotype, EMT-like morphological changes, loss of epithelial cell polarity, and junctional defects), (4) resistance to cell death (p53 inactivation and MCL1 induction), and (5) genome instability and mutability (AID induction and mitotic defects). The remaining hallmark, (6) induction of tumor-promoting inflammation (NF-κB/inflammasome activation), is induced non-cell-autonomously (Fig. 2) (Table 1).

**Table 1:** Pro-oncogenic effects of *cagA*-positive *H. pylori* infection.

	Related cancer hallmarks	Pro-oncogenic actions	Molecular mechanisms	CagA dependency	Responsible factors	Reference no.
Cell autonomous pro-oncogenic actions	Sustained proliferative signaling	RAS-ERK signaling activation	Deregulation of SHP2	pY-CagA	EPIpYA-C/EPIpYA-D	^90^
		Wnt signal activation	Complex with E-cadherin	CagA	CM motif	^112, 113^
			Activation of cMet-PI3K-AKT signaling			^114^
			Sequesteration of GSK3β		C-terminal region	^115^
			Degradation of RUNX3		−	^116, 117^
	Growth suppressors	Degradation of RUNX3	−	CagA	−	^116, 117^
		Degradation of p53	Complex with ASPP2		N-terminal region	^124, 126^
			Activation of HDM2 and ARF-BP1		−	^127, 128^
		Inactivation of p53	Ectopic expression of AID			^130^
	Activate invasion and metastasis	Induction of hummingbird cell	Deregulation of SHP2	pY-CagA	EPIpYA-C/EPIpYA-D	^70, 85, 91, 92^
		Perturbation of focal adhesion dynamics	Dregulation of SHP2-FAK signaling			^91^
		Loss of apicobasal cell polarity	Inhibition of PAR1b	CagA	CM motif	^104, 123^
		Tight junctional defect	Inhibition of PAR1b			^104^
		Adherence junctional defect	Complex with CRK	pY-CagA	Tyrosine phosphorylation	^120^
		Altered stress fiber formation	Inhibition of PAR1b-GEF H1 signaling	CagA	CM motif	^110^
		Induction of transdifferentiation	Ectopic expression of CDX1 and CDX2		−	^118, 119^
		EMT-like morphological change	Junctional and apicobasal polarity defects			^121–123^
	Cell death	Resistant to apoptosis	Complex with ASPP2	CagA	N-terminal region	^124, 126^
		Promoting cell survival	Activation of MEK-ERK-MCL1 signaling		−	^134^
	Genomic instability and mutation	Mitotic defects	Inhibition of PAR1b	CagA	CM motif	^109^
		Hyper mutated phenotype	Ectopic expression of AID		−	^130^
			CpG hypermethylation of *MGMT*	−	Potentiated by *cagA*	^133^
		Induction of DNA double strand break	Inhibition of PAR1b	CagA	CM motif	^131^
			−	*−*	Potentiated by *cag*PAI	^132^
Non-cell-autonomous pro-oncogenic actions	Induction of tumor-promoting inflammation	Sensitizing to NF-κB activation	Reduction of IκB in cells	CagA	CM motif	^166^
		Activation of NF-κB	−		−	^130^
			IKK activation via cMet-PI3K-AKT signaling		CM motif	^114^
			IKK activation via TAK1-TRAF6 signaling		−	^169^
		Induction of IL-1β	Inflammasome activation in epithelial cells			^166^
		Induction of IL-8	Activation of PI3K	pY-CagA	EPIpYA-B	^148^
		Activation of STAT3	IL-6/gp130 signaling	CagA	pY independent	^170^
		Activation of NF-κB	Activation of Nod1	*−*	Peptidoglycan	^171, 172^
			Activation of ALPK1-TIFA signaling		ADP heptose	^174–176^
			Activation of TLR2, TLR4 signaling		Lipopolysaccharide	^173^
			Activation of NLRP3-inflammasome		*cag* PAI	^177–179^

**Fig. 4:**
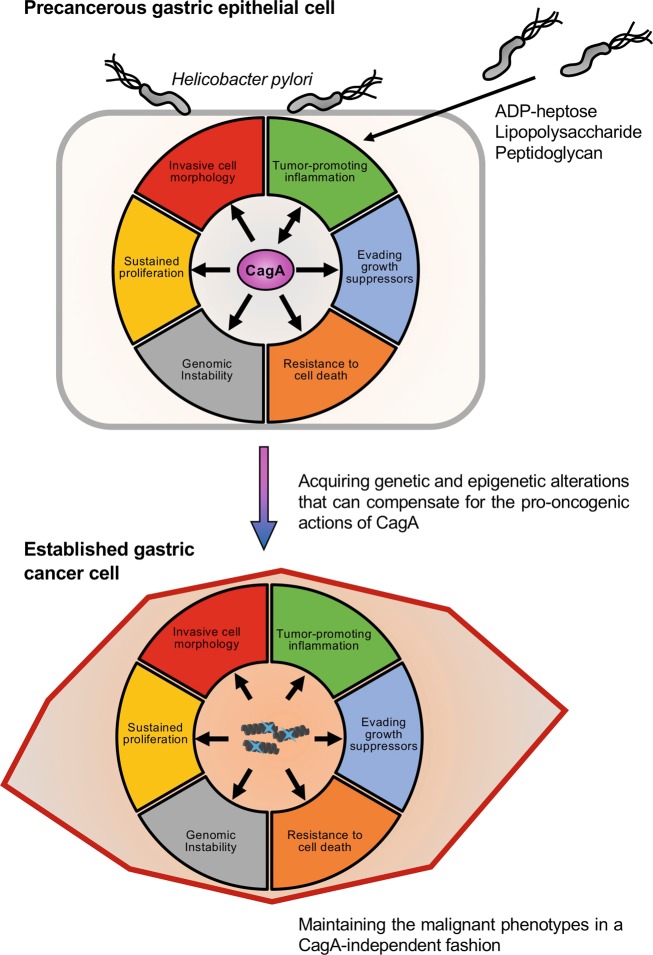
Schematic representation of *H. pylori* CagA-directed “hit-and-run” gastric carcinogenesis. When delivered into gastric epithelial cells, the *H. pylori*-derived CagA oncoprotein perturbs multiple intracellular signaling pathways, which promotes malignant transformation of the host cells. There is a mutual feedforward stimulatory mechanism between the oncogenic activities of CagA and the pro-inflammatory responses against *cagA*-positive *H. pylori* infection. The direct priming of pro-oncogenic signaling by CagA in the precancerous stage promotes the acquisition of genetic and epigenetic alterations that can compensate for the perturbed cell signaling by CagA. Therefore, the established gastric cancer cells no longer require CagA protein to maintain their malignant phenotypes.

Spontaneous development of gastrointestinal neoplasms in *cagA*-Tg mice in the absence of infiltrating immune cells indicates that prolonged CagA expression is sufficient for carcinogenesis in this particular transgenic system.^157^ This suggests that inflammatory responses are not indispensable for CagA-induced gastrointestinal carcinogenesis, which may depend on mutual feedforward augmentation of the five malfunctional biological processes in CagA-expressing cells. This notion is consistent with the observations in *claudin18*-null (*Cldn18*^−/−^) mice, a mouse model of chronic atrophic gastritis that develops neither dysplasia nor carcinoma in the stomach.^180^ However, along with the well-recognized notion that chronic inflammation fosters carcinogenesis, inflammation indeed strengthens the oncogenic potential of the CagA protein.^165,166^ Although it remains unclear to what extent CagA *per se* is involved in *H. pylori*-induced gastritis, the oncogenic actions of CagA and *cagA*-positive *H. pylori*-induced inflammation reinforce each other in the progression of gastric carcinogenesis (Fig. 4).

Inconsistent with the well-recognized pivotal roles of *H. pylori* infection in gastric carcinogenesis, established gastric cancer cells maintain their transformed phenotypes in the absence of *H. pylori* colonization. Therefore, during the process of multistep gastric carcinogenesis, CagA-injected gastric epithelial cells must acquire genomic and epigenomic alterations that can compensate for the pro-oncogenic functions of the CagA protein. This “hit-and-run” hypothetical process of *cagA*-positive *H. pylori*-induced gastric carcinogenesis is thought to be achieved by the induction of genomic instability due to the ectopic expression of AID and/or the mitotic defects caused by CagA. The CagA-induced genetic mutability, which occurs concomitantly with the acquisition of resistance to cell death, enables the cells to accumulate genetic alterations. Aberrantly regulated cell cycle progression occurring via the tyrosine-phosphorylated CagA-SHP2-ERK axis, which is influenced by CagA polymorphisms, may accelerate the process of AID-dependent and/or mitosis-dependent acquisition of genetic alterations^181^ (Fig. 4). Genetic and epigenetic alterations frequently found in gastric cancers, including amplification of genes involved in RAS-ERK signaling (*KRAS*, *FGFR2*, *EGFR*, *ERBB2*, and *MET*),^182^ gain of function of the *CTNNB1* gene (encoding β-catenin),^183^ and loss of function of the *TP53* gene (encoding p53) and the *CDH1* gene (encoding E-cadherin),^184–187^ may play key roles in the functional compensation of CagA-directed cancer hallmarks.

An in vivo lineage tracing study in a mouse stomach indicated that the adult pyloric epithelium self-renews within two weeks.^188^ Therefore, to contribute to the initiation and progression of the multistep “hit-and-run” carcinogenesis in the gastric epithelium, *H. pylori* must adhere to and subsequently deliver CagA into long-lived stem/progenitor cells. Regarding this issue, direct contact of *H. pylori* with the stem/progenitor cell population, which is defined as either Lgr5+/Axin2+ cells or Lgr5−/Axin2+ cells, has been shown in human/mouse gastric glands. Furthermore, *H. pylori* infection of stem/progenitor cells causes glandular hyperplasia associated with an expansion of the cells with Lgr5 expression and/or Axin2 expression upon the function of  T4SS, which delivers CagA into the cells.^189,190^ Additionally, since both *Lgr5* and *Axin2* are known to be specific target genes of Wnt signaling, which plays an indispensable role in the maintenance and self-renewal of epithelial stem cells, deregulation of Wnt signaling by CagA may promote the expansion of stem/progenitor cells, which may accelerate the progression of “hit-and-run” gastric carcinogenesis.

## Conclusions and future perspectives

*H. pylori* CagA is the first identified bacterial oncoprotein that hijacks multiple cellular processes associated with carcinogenesis. The functional interplay that mutually occurs and accelerates the CagA-induced cell-autonomous pro-oncogenic actions is thought to be fundamentally required for the generation of the “hit-and-run” circuit that drives the acquisition of genetic alterations for multistep carcinogenesis. Additionally, mutual reinforcement between the pro-oncogenic actions of CagA and *H. pylori*-induced inflammation adds another layer of oncogenic interplay by further accelerating the acquisition of genetic alterations that eventually generate cells with the cancer hallmark phenotypes in a CagA-independent manner (Fig. 4). In several previous experiments, oncogenic stress induced by CagA provoked premature cell senescence [oncogene-induced senescence (OIS)] through aberrant induction of the cell cycle inhibitor p21.^112,123,191^ Therefore, the acquisition of genetic alterations that can overcome the CagA-induced OIS may also be a key step in *cagA*-positive *H. pylori*-induced “hit-and-run” gastric carcinogenesis.

In addition to the interaction between *H. pylori* and host cells, a functional interplay between CagA and other virulent proteins of *H. pylori* [*e.g*., vacuolating cytotoxin A (VacA)] may further modulate the oncogenic actions of CagA.^82,191^ Moreover, since sustained tyrosine phosphorylation of CagA is induced via *SHP1* suppression in EBV-coinfected gastric epithelial cells, colonized *cagA*-positive *H. pylori* may modulate and benefit from the composition of the gastric mucosal microbiota, suggesting a yet-to-be-known extra layer of interplay: mutual modulation between the oncogenic actions of CagA and the gastric mucosal microbiota.^76,192,193^ Although the biological relevance of bacteria other than *H. pylori* in the stomach needs to be further investigated, a functional interplay between *cagA*-positive *H. pylori* and the gastric microbiota, which includes EBV, may provide better insight into understanding the mechanism of CagA-induced “hit-and-run” gastric carcinogenesis.

*H. pylori* infection is crucial for gastric carcinogenesis, whereas *H. pylori* is no longer  required in established gastric cancer. The concept of CagA-directed “hit-and-run” carcinogenesis provides insights for a better understanding of this paradoxical phenomenon by suggesting a dual oncogenic action of the CagA protein: priming of pro-oncogenic signaling pathways and promotion of genomic instability. Further investigation is needed to evaluate the relevance of the hypothetical concept.
